# Hidden Markov models reveal complexity in the diving behaviour of short-finned pilot whales

**DOI:** 10.1038/srep45765

**Published:** 2017-03-31

**Authors:** Nicola J. Quick, Saana Isojunno, Dina Sadykova, Matthew Bowers, Douglas P. Nowacek, Andrew J. Read

**Affiliations:** 1 Division of Marine Science and Conservation, Nicholas School of the Environment, Duke University, Beaufort, North Carolina 28516, USA; 2 School of Biology, University of St Andrews, Bute Building, St Andrews, Fife KY16 9TS UK; 3 Zoology School of Biological Sciences, University of Aberdeen, Tillydrone Ave, Aberdeen, AB24 2TZ, UK; 4 Electrical and Computer Engineering, Pratt School of Engineering, Duke University, Durham, NC 27708, USA

## Abstract

Diving behaviour of short-finned pilot whales is often described by two states; deep foraging and shallow, non-foraging dives. However, this simple classification system ignores much of the variation that occurs during subsurface periods. We used multi-state hidden Markov models (HMM) to characterize states of diving behaviour and the transitions between states in short-finned pilot whales. We used three parameters (number of buzzes, maximum dive depth and duration) measured in 259 dives by digital acoustic recording tags (DTAGs) deployed on 20 individual whales off Cape Hatteras, North Carolina, USA. The HMM identified a four-state model as the best descriptor of diving behaviour. The state-dependent distributions for the diving parameters showed variation between states, indicative of different diving behaviours. Transition probabilities were considerably higher for state persistence than state switching, indicating that dive types occurred in bouts. Our results indicate that subsurface behaviour in short-finned pilot whales is more complex than a simple dichotomy of deep and shallow diving states, and labelling all subsurface behaviour as deep dives or shallow dives discounts a significant amount of important variation. We discuss potential drivers of these patterns, including variation in foraging success, prey availability and selection, bathymetry, physiological constraints and socially mediated behaviour.

Cetaceans live most of their lives underwater and thus engage in a great variety of subsurface behaviour. Classification of any repertoire of diving behaviour requires identification of objective criteria that allow an observer to discriminate among various dive types; several methods have been used to identify such categories in odontocetes, see ref. 1 for review. These methods range from subjective grouping of dives based on a certain characteristic (e.g. maximum depth) to the more objective use of statistical techniques. Analysis of diving in cetaceans has been improved by the development of animal-borne tags that provide high resolution data on kinematic and acoustic behaviour^2^. However, analysing complex, non-independent, time series data and quantifying the likelihood of an individual type of behaviour based on a series of observed data points from a tag record presents a particular set of challenges. Inherent differences among individual animals, motivational states and environmental factors may all contribute to observed behaviour. Furthermore, it is difficult to scale up from an individual tag record to a population-level behavioural model. The use of non-invasive digital acoustic recording tags (DTAGs), attached via suction cups^3^, has provided detailed records of diving behaviour in a number of deep-diving cetaceans, including sperm whales *Physeter macrocephalus*^4^, beaked whales^5^,^6^,^7^, short-finned pilot whales *Globicephala macrorhynchus*^8^,^9^ and long-finned pilot whales *Globicephala melas*^10^,^11^. DTAGs record kinematic and depth measurements of subsurface behaviour, together with a synchronized acoustic record, providing a rich data series of variables that can be associated with diving behaviour.

Pilot whales are social toothed whales found world-wide in tropical and sub-tropical waters of the shelf break and continental slope^12^. Two species exist, and both (long-finned and short-finned) are capable of performing foraging dives to hundreds of meters^8^,^9^,^13^,^14^,^15^,^16^,^17^. Temporal clustering, or bouts, of dives has been suggested for long-finned pilot whales, with periods of shallow diving followed by bouts of deep diving^10^,^11^. For short-finned pilot whales, dive records are also suggestive of temporal clustering, but no information exists about transitions between diving states or time activity budgets, nor how these may be affected by the duration of tag deployment.

In the four previous studies of diving behaviour that employed DTAGs, diving was, in the first instance, defined by maximum depth values. For short-finned pilot whales, Jensen *et al*.^9^ defined deep dives as submergences exceeding 300 m. Aguilar de Soto *et al*.^8^ initially scored a dive as any submergence of more than 20 m and further discriminated between shallow and deep dives using a threshold of 500 m. For long-finned pilot whales, Sivle *et al*.^10^ and Visser *et al*.^11^ used a log frequency analysis to define a cut-off of 34 m to separate shallow and deep dives. Dives were then further classified as foraging dives by other metrics including the presence of vocal behaviour (click trains and buzzes, associated, although not exclusively, with foraging^8^,^9^) similar to those seen during foraging in other deep diving odontocete cetaceans, such as beaked whales and sperm whales^4^,^5^, and short-finned pilot whales^8^, or kinematic measures such as sprints^8^.

Diving behaviour in pilot whales may approximate an optimal solution to the trade-off of maximizing foraging time versus available oxygen stores, as has been suggested for other diving vertebrates^18^,^19^. However, Aguilar de Soto *et al*.^8^ demonstrated a high risk-high gain strategy employed by short-finned pilot whales off Tenerife, in which whales engaged in daytime foraging targeting large, high-value, fast moving prey such as giant squid. These observations are in contrast to the predictions of simple optimal foraging theories of maximizing time at depth based on the scaling relationship between mass and metabolic rate alone, and are more in line with suggestions that prey quality may shape foraging strategies^20^. The only study of the stomach contents of mass-stranded pilot whales off Cape Hatteras, North Carolina, USA, revealed a diet of small-bodied mesopelagic squid^21^, which are unlikely to be of high energetic value, suggesting a different foraging strategy to that documented by Aguilar de Soto *et al*.^8^. Variation in foraging depths of pilot whales has been suggested as an adaptation to the diel migration of prey, with foraging dives to shallower depths during night time^8^,^13^. Pilot whales off Cape Hatteras, do not show any discernible diurnal pattern in dive behaviour, with no significant diel differences in the number dives per hour or mean maximum depth of dives (M. Bowers 2016, Unpublished PhD Thesis, Duke University). They also show no patterns in dynamic acceleration or sprinting with depth (M. Bowers 2016, Unpublished PhD Thesis, Duke University) in contrast to that seen in other populations^8^. Similarly, the existence, albeit rare, of buzzes during relatively shallow dives (Ref. 8, M. Bowers 2016, Unpublished PhD Thesis, Duke University) suggests complexities in pilot whale diving behaviour that may not be captured by a simple dichotomy of deep foraging and shallow, non-foraging diving states.

In the present study we use multi-state hidden Markov models (HMMs) to classify dives into one of N unobserved states in short-finned pilot whales, based on DTAG data. We use three observed parameters, that we consider predictors of dive type, to assign the most likely state and the transitions between them.

## Results

The 20 deployments produced 124 hours and 6 minutes of recording time from 20 different whales (Table 1). The tags were deployed for periods that varied from 0.5 to over 18 hours (median 3 hours, 10 minutes), and the number of dives per individual ranged from 2 to 64 (Table 1). Data were not distributed evenly through day and night; all tags were attached during daylight hours. Twelve of the twenty tags recorded only during daylight hours, with tag off times prior to 18:00 hours. Two other tags released prior to 20:30 during summer months, when daylight was reduced. A further two released after 22:30 but prior to midnight, and four tags remained on overnight, releasing at approximately 06:00 or later the following morning (Table 1). Sex was determined for nine individuals, and included three females and six males (Table 1).

### State Allocation

**Figure 1:**
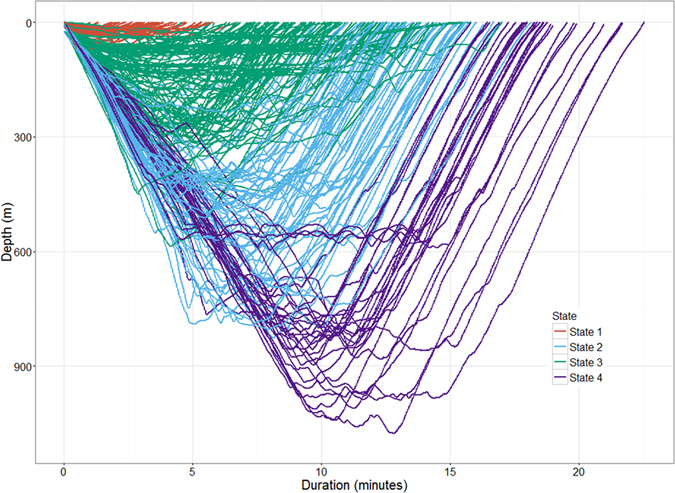
Dive allocation per state for all dives from all individuals. Red = state 1, blue = state 2, green = state 3, purple = state 4.

**Figure 2:**
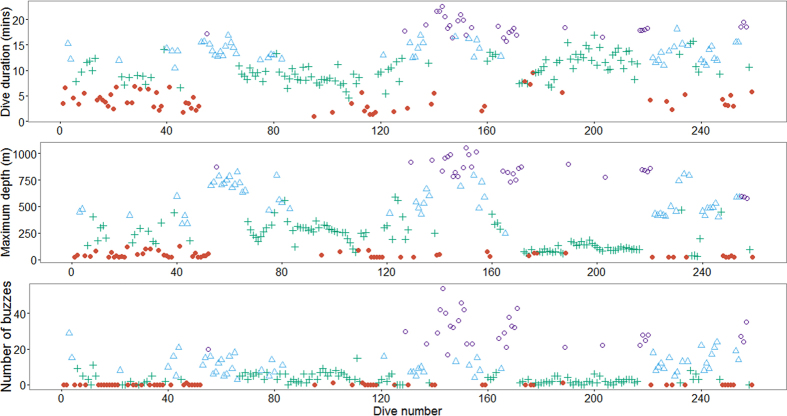
State allocation per dive for each of the three observed variables. Red filled circles represent state 1; blue triangles represent state 2; green crosses represent state 3 and purple open circles represent state 4. All dives for all individuals are shown sequentially in chronological order along the X-axis.

**Figure 3:**
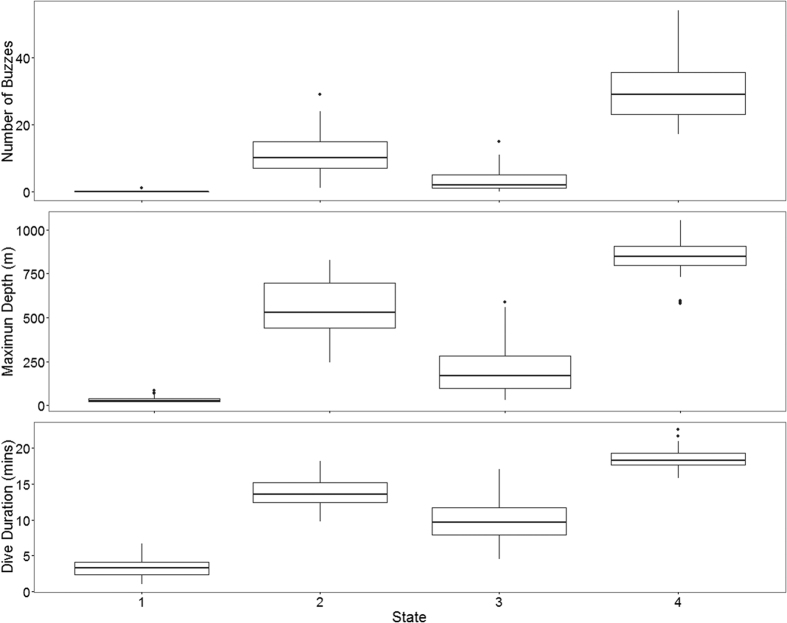
Box-plots for each of the three variables for all four states, showing median value, 25^th^ and 75^th^ percentiles, min-max range and outliers.

**Figure 4:**
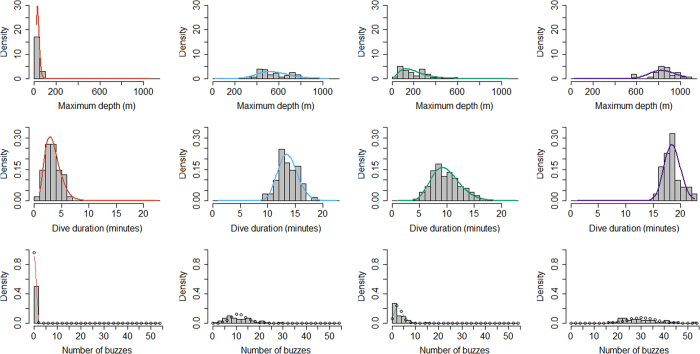
Fitted state dependent distributions and observed data for the best model.

The four state hidden Markov model (HMM) with no random effects, consistently produced better AIC and BIC scores than the other nine models (Table S1). Re-running of all models with different initial values showed stable AIC and BIC scores and negative log-likelihood values, and consistent state allocation of all dives across all individuals. We interpret the four states to represent different types of diving behaviour in short-finned pilot whales. State 1 included shallow dives, characterized by short durations and no buzzes. State 4 dives were deep and long with a very high buzz rate. State 2 and state 3 were intermediate between state 1 and state 4 dives, with state 3 dives being on average shallower, shorter and with fewer buzzes than state 2 dives (Figs 1, 2, 3). Analysis of each observable data series by dive state (Fig. 2) shows overlap between the four states and variation between dives within each state (Figs 2 and 3). Plots of the state-dependent distributions against the observed data are shown in Fig. 4. Overlap in variable range is seen between many of the states (Figs 2 and 3), particularly states 2 and 3, which overlapped considerably for each variable. State 1 had the narrowest range for two variables (Fig. 3) and no variable range overlapped with state 4. In state 1 all but four (out of 59 dives) dives contained no buzzes (Figs 2 and 3) and dives were generally shallow (median depth 27 m) and short (median 3.33 minutes). The four dives in state 1 that contained a buzz had maximum depths of 60 m, 60 m, 74 m and 87 m and durations of 3.6, 5.6, 1.8 and 5.7 minutes, comparable in depth and duration to all other dives in the state. For state 2, the shallowest dive (245 m) had a just below average dive duration (12.8 mins) and number of buzzes (9). In state 3, two dives of long duration (15 minutes, state median is 9.6 minutes) with average and high buzz number (3 and 8 buzzes, mean buzzes is 2) were the shallowest in the state, both less than 40 m deep (state 3 median depth was 171 m). For state 4, the shallowest dives in the state had longer than average durations and an average number of buzzes for the state (Figs 2 and 3).

### Time Budgets

**Figure 5:**
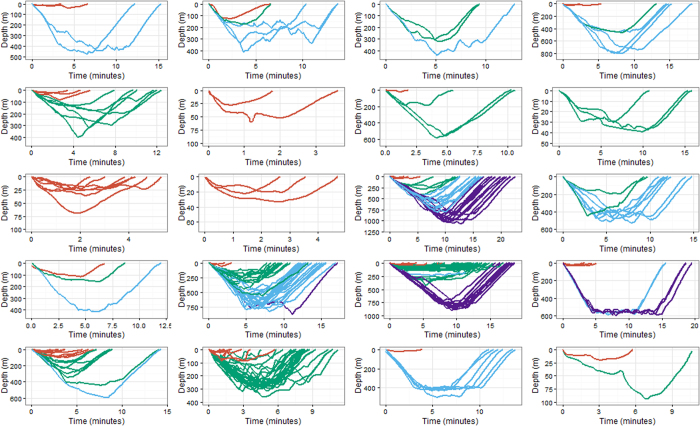
Dive allocation per individual (a single tag record each). Red = state 1, blue = state 2, green = state 3, purple = state 4. See Table 1 for individual information; First column, individuals 143a-151b; second column, individuals 185b-208a; third column, individuals 209a-149b; fourth column, individuals 150b-280a Note different x and y axis scales.

**Figure 6:**
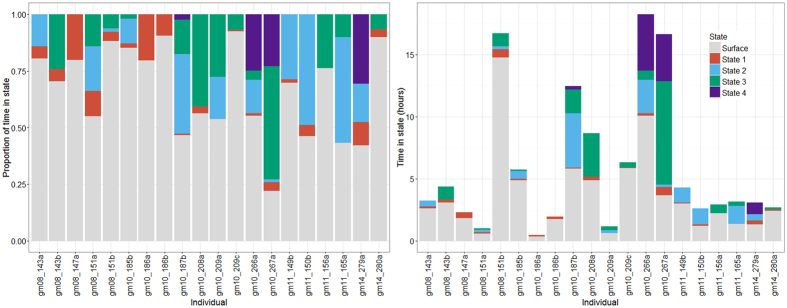
Time budgets for each individual whale. Left panel shows proportion of time in state. Right panel shows actual time in state for each tag duration. Grey = surface (i.e. data not used in the analysis, including shallow dives classified at surface behaviour (max depth < 20 m)), red = state 1, blue = state 2, green = state 3, purple = state 4.

There was considerable variability in the prevalence of dive types both within and across tag records. Of the 259 dives, the shallowest dive type (state 1) was allocated to 59 dives across most (17) individuals; state 2 was allocated to 57 dives across 12 individuals; state 3 was the most common dive type (112 dives across 14 individuals) while state 4 was allocated to the fewest number of dives and individuals (31 dives across only 4 individuals) (Table 1, Fig. 5). Four whales displayed a single state; eight displayed two states; five displayed three states and three displayed all four states (Fig. 5). All four states occurred during both day and night (Fig. 6). None of the three known female whales displayed any state 4 dives (Table 1), but they displayed all other dive types. The six known males displayed all dive types, with two showing state 4 dives. Time budgets were calculated for each individual whale and showed that the proportion of time spent diving and time in different diving states varied across individuals (Fig. 6). Fifteen whales spent greater than half of the time at the surface (defined as shallower than 20 m) and nine spent greater than 75% of the time at the surface (Fig. 6). There was no linear relationship between the duration of the tag record and the proportion of time spent at the surface (*R*^2^ = *0.073, F (1, 18*) = *1.427, p* = *0.248*). However, the model was estimated to have relatively low power to detect such a relationship (0.7 for an effect size of 0.35 at 5% significance level). The multiple regression showed no significant relationship between tag duration and the occurrence of one or two or one or three states, but did show a significant relationship between tag duration and exhibiting one or four states (*R*^2^ = *0.7157, F (3, 16)* = *13.42, p* < *0.001*). The model was estimated to have low power (0.48 for an effect size of 0.35 at 5% significance level) (Supplementary Fig. S1 and Supplementary Table S2).

### State Transitions

**Figure 7:**
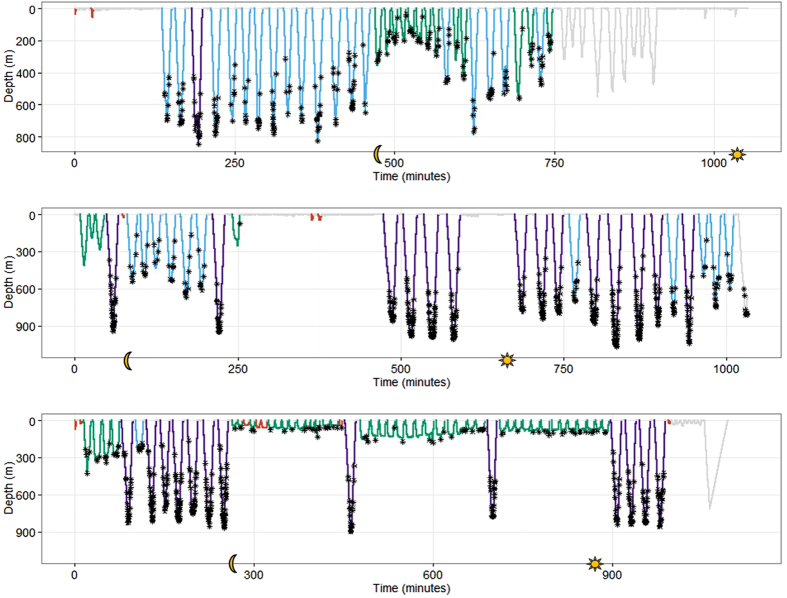
Dive profile data from three individuals (top: gm10_187b, middle: gm10_266a, bottom: gm10_267a) with the most likely state sequences mapped onto the dives. Red lines indicate highest probability of being in state 1, blue lines indicate highest probability of being in state 2, green lines indicates highest probability of being in state 3 and purple lines highest probability of being in state 4. Grey lines indicate data not used in the analysis, including shallow dives classified at surface behaviour (max depth < 20 m), and incomplete dives or dives lacking acoustic records. Black asterisk identify individual buzzes, yellow moon on x axis indicates time of sunset (20:00) and yellow sun indicates time of sunrise (6:00). Note different x and y axis ranges.

State persistence and state switching were observed within and across all states. The transition probabilities were considerably higher for state persistence than state switching for all states, indicating that dive types occur in bouts (Table 2 and Fig. 7). The highest transition probabilities were seen for persistence in states 2 and 3 and the lowest in state switching between state 3 to state 4 and state 1 to state 4 (Table 2). Mapping the most likely (Viterbi) state sequences onto the dive profiles supports the existence of bouts, i.e. state persistence, but also shows examples of state switching (Fig. 7). There was a higher proportion of state 1 and state 3 dives than would be expected (0.25) if all dives appear equally, supporting the existence of clusters of dives. Mean proportion of state 1 is 0.355 (median: 0.275, Interquartile range: 0.105–0.5, *N* = 20), of state 2 is 0.256 (median: 0.129, Interquartile range: 0–0.507, *N* = 20), of state 3 is 0.340 (median: 0.278, Interquartile range: 0–0.667, *N* = 20), and of state 4 is 0.049 (median: 0, Interquartile range: 0–0, *N* = 20).

## Discussion

Our findings support the conclusion that the diving behaviour of short-finned pilot whales is more complex than a simple dichotomy of deep and shallow dives. The application of the HMM suggests that subsurface behaviour of pilot whales off Cape Hatteras can be classified into four dive types or diving states. The four diving states were observed across multiple whales and provided insight into patterns of state persistence and state switching. Our predictions of diving state are based on the multivariate distributions of three readily observed variables. We chose dive duration, maximum dive depth and number of buzzes as good descriptors of diving behaviour based on previous studies of this species^8^,^9^,^16^. We also drew from an analysis of data from pilot whales off Cape Hatteras that showed dive duration and maximum depth to be the two most important predictors of foraging dives, with kinematic variables such as overall dynamic acceleration or average speed of movement showing no strong pattern with depth (M. Bowers 2016, Unpublished PhD Thesis, Duke University). Most prior studies have relied on depth to define two states/types (deep and shallow) of dives. Furthermore, as noted by Alves *et al*.^16^, a variety of depths have been used to differentiate shallow and deep dives in pilot whales^8^,^9^,^10^,^11^,^13^. The HMM allowed us to classify diving behaviour more objectively using relevant observational variables, whilst accounting for the autocorrelation in the time series data and computing the most likely state sequence and the transitions between states.

An HMM framework is commonly applied to animal movement data to infer drivers of behaviour with the aim of connecting the estimated states to distinct behavioural modes. In our study we use HMMs to estimate the number of distinct dive states. Previous studies have noted that a one-to-one correspondence between nominal HMM states and biologically meaningful behavioural states is not necessarily guaranteed^22^,^23^,^24^,^25^. Our study aimed to estimate dive types through exploring the variation in three variables taken from individual dives. As pilot whales dive to forage and buzzes are commonly used as proxies for foraging behaviour in cetaceans^4^,^5^,^8^,^9^ the biological relevance of our states is skewed towards modes within this behavioural class. We interpret state 1 to be non-foraging behaviour and states 2–4 to represent different modes of foraging behaviour, that imply different amounts of depth driven foraging, as measured by the number of buzzes. From a biological perspective, the split of dives between these three states is made not only from distinct differences in terms of maximum depth and duration, but also on the magnitude of foraging effort. This approach has been used previously for blue whales with shallow foraging and deep foraging, as measured by the number of lunges, specified as separate states within a HMM framework^25^.

The choice of a four state model was based on numerical stability and model selection criteria. The four state model may have been selected over the two or three state models to compensate for an otherwise overly simplistic model formulation that does not adequately account for substantial heterogeneity between individuals. However, for three and four state models, the incorporation of individual variability through random effects did not select for the more complex models, instead choosing the less complex models assuming no heterogeneity (Supplementary Table S1). This may be due to our data set not containing long enough time series to truly detect individual variability and estimate the extra parameters for a random effect. It is also possible that random effects are only one expression of variability and there may be greater temporal dependence than a first order Markov model. Not all individuals displayed every state, but the states were distributed across multiple individuals suggesting that they are broadly comparable across individuals.

All four states showed temporal clustering, indicating that these states occur in bouts. There was no significant difference between tag duration and the occurrence of one or two states, or one or three states, but there was a significant difference in tag duration and displaying one or four states. State persistence, for all states, was shown by some individuals for over 200 minutes, suggesting that short duration tag deployments may present only a snapshot of an individual’s diving behaviour. The five whales monitored for the shortest periods (less than 2.5 hours, Table 1) displayed only one or two states, in contrast to the six animals tagged for the longest periods (greater than 6 hours, Table 1) which exhibited two, three or four diving states.

There are no prior studies of time activity budgets of short-finned pilot whales, but studies from other social cetacean species demonstrate that individuals often engage in bouts of behaviour and rarely behave in a sequentially random fashion^26^. It is, therefore, possible that the longer the monitoring period, and resulting time series of data, the higher the probability of recording all diving states, if all individuals are equally likely to engage in all states. Based on the proportion of dives in each state, it is clear that pilot whales tend to dive in clusters of like dives. However, it is difficult to predict whether every individual will engage in all four states without understanding the mechanisms that drive the differences among states and determining which behaviour is represented by each state. The time activity budgets of the twenty pilot whales in this study showed considerable variation in the proportion of time spent in different states. (Fig. 6). Tag duration was not a good predictor of surface time but, in general, shorter duration tags (less than 5 hours) tended to have a single dominant diving state, supporting the existence of bouts of behaviour, perhaps driven by the whale targeting a specific prey space in consecutive dives.

State 1 was characterized by shallow dives, short durations and, for all but four dives, an absence of foraging attempts (buzzes). The mean depth value (43 m) was similar to the cut-off value used for defining shallow and deep diving states in long-finned pilot whales^10^,^11^. State 1 dives were observed in multiple individuals, with a higher probability of state persistence than state transition, suggesting they represent a defined behaviour or group of behaviours. We consider this state to represent non-foraging behaviour, but we are unable to conclude a specific function for this state from our data, and suggest that these dives could serve either a social or drag reduction function. Short-finned pilot whales are highly vocal animals^27^, and rely on acoustic communication to maintain social bonds. The increased propagation of sound underwater compared to in air^28^ suggests pathways for acoustic communication will be maximized subsurface. Similarly, a reduction in surface drag to aid efficient movement, see ref. 29 for review, suggests more efficient locomotion could be performed subsurface, although many of the state 1 dives may be too deep for horizontal travel to be their primary function. State 1 dives could, therefore, represent either (or both) of these behaviours. We are currently using observations of non-foraging behaviours, such as social call rates, or speed of movement, to investigate the function of these dives.

There are a number of plausible explanations that could underlie the observed variation in diving behaviour seen in states 2–4, including physiological limitations, prey selection and abundance, geographic topography and socially mediated behaviour. Physiological limitations in diving ability limit the maximum depth and duration of a dive. Greater body size affords an increase in oxygen stores, allowing larger animals to dive for longer and deeper^30^. Short-finned pilot whales are sexually dimorphic^12^, with adult females typically ranging between 3–4 m^31^ and adult males between 4–5 m in length^32^. State 4 dives are longer and deeper than other dives and perhaps represent the physiological limit of the diving ability of this species off Cape Hatteras. Mori^33^ suggests that individuals of different body sizes occupy different depths when feeding on patchy prey. However, we do not have complete sex or age class information for all individuals sampled, so it is not possible to fully address this question, and only one of the six tags with longest duration (greater than 6 hours) was deployed on a female whale. However, this female, despite over 8 hours of tagging data, and with 34 dives, did not undertake any state 2 or state 4 dives and her deepest dive was only to 362 m (Table 1 and Fig. 5). Individual short-finned pilot whales in Tenerife demonstrate a high-risk, high-gain strategy to target large, fast moving prey during daytime dives^8^. The maximum depths and dive durations seen in Tenerife are very similar to those in our state 2 and state 4 dives, but the number of buzzes is strikingly different and we recorded no sprints, as described by Aguilar de Soto *et al*.^8^, in our data records (M. Bowers 2016, Unpublished PhD Thesis, Duke University). The mean number of buzzes for state 2 off Cape Hatteras was 11.65 and for state 4 was 30.45, compared to 1.5 for the Tenerife animals^8^. This suggests that pilot whales off Cape Hatteras either attempt to capture multiple prey items, perhaps of lesser calorific value, during the state 2 and 4 dives, unlike the foraging behaviour observed off Tenerife^8^, or buzzes are indicative of something other than simple prey capture attempts.

Optimal diving theory predicts that individuals should maximize foraging, subject to the constraints of oxygen stores^34^. In state 4 dives, large pilot whales may maximize the duration of time spent foraging, increase the number of prey encounters and dive to greater maximum depths. However, it is clear that short-finned pilot whales off Cape Hatteras employ a variety of diving strategies, suggesting that physiology and/or simple optimal diving rules do not always drive diving behaviour. Short-finned pilot whales off Cape Hatteras exploit a wide range of food types, with a predominance of oceanic deep water squid^21^. Their diving ability enables them to exploit the epipelagic, mesopelagic and benthic habitats, suggesting prey selection and relative abundance could also be driving the variation we observed here. There is no single solution to the problem of exploiting mobile aquatic prey and individual whales likely vary their strategy dive by dive^35^. We have no measure of success during prey capture attempts, but the variation in number of buzzes could reflect a simple increase in the number of buzzes to compensate for increased transit times during deeper dives, multiple failed foraging attempts or selection of prey items of different sizes and perhaps calorific value. Shorter dives during states 2 and 3 may indicate success earlier in the dives, negating the need to dive deeper, or represent animals breaking off from dives due to limited foraging success. In this latter case we would predict that animals would switch diving states to target a more profitable prey layer. However, the probability of state persistence, for all states, was considerably higher than state transitions, indicating that individuals had a tendency to continue a foraging strategy even when it constituted shallower dives.

Individual foraging specializations have been observed in a number of marine mammals including bottlenose dolphins, *Tursiops.spp.*^36^, Antarctic fur seals, *Arctocephalus gazella*^37^, grey seals, *Halichoerus grypus*^38^, narwhals, *Monodon monoceros*^39^ and sea otters, *Enhydra lutris nereis*^40^. Individual pilot whales may also show disparities in prey preference, foraging success or foraging specializations, that could contribute to the variation we observed in diving behaviour, but without data on actual prey availability and consumption it is impossible to determine if individual specialization is a key driver of the observed differences in diving. Diving behaviour in other pilot whales has been shown to vary with diurnal cycle in response to vertically migrating prey, with deepest dives being made during the day^8^,^13^,^17^. In contrast, analysis of long term satellite-linked time-depth recorders (SLTDR tags) attached to six individual short-finned pilot whales off Cape Hatteras, North Carolina showed no diel patterns in foraging rates or foraging depths (M. Bowers 2016, Unpublished PhD Thesis, Duke University). During our study, all four of the individuals that were tagged overnight, displayed a range of diving states (see Fig. 6 for two examples) with no evident diel patterns.

The variation we observed in diving behaviour could also be explained, in part, by the topography of the area. The slope area of Cape Hatteras has steep bathymetric gradients^41^ and all DTAGs were deployed on animals in this area. We do not have fine-scale positional data for each dive, but the differences in foraging depth could be driven by bottom topography. Other studies of pilot whale diving behaviour have shown animals foraging at the extents of the bathymetry^15^ and this could explain some of the state allocations across individual whales. In general, pilot whales are not considered to forage exclusively on benthic prey, however, and their dive shapes do not consistently follow the typical U-shaped patterns seen by diving animals that feed in this manner (e.g. ref. 42).

Pilot whales are highly social animals, which are thought to live in long-term stable groups^43^ and perform highly synchronous surface behaviour^44^. Social foraging and the linkages between individual and emergent group-level time budgets has been studied in a range of species (e.g. ref. 45) including long-finned pilot whales, in which whales from the same social group coordinate their foraging behaviour^11^. In this social foraging strategy, group members synchronize diving bouts, but do not synchronize each individual dive. This may aid in maintaining social group cohesion, especially in areas where large numbers of individual animals are present, such as our study site where dense aggregations of pilot whales occur along the shelf break^46^. As a consequence, the need for social cohesion may dictate individual diving behaviour, with animals making group-based decisions to forage^47^. Our non-systematic visual observations show that individuals within a single social group often synchronise their surface behaviour and timing of dives. We also have data from a single simultaneous tagging event of two whales that demonstrated synchronous diving behaviour (M. Bowers 2016, Unpublished PhD Thesis, Duke University). There may also be constraints imposed by the need of females to care for small calves with limited diving abilities. Sperm whale groups have been shown to alter dive behaviour when a calf is present, with an adult more frequently observed at the surface compared to sperm whale groups without calves^48^. None of our tagged individuals had noticeable dependent calves, but all tagged animals were in social groups.

The higher probability of state persistence than state transitions suggests that each state is indicative of a bout of behaviour. We observed transitions between all states, indicating that individuals switch between different states. A recent study of sperm whale foraging behaviour demonstrated how these whales switch between different prey layers in successive foraging dives based on acoustic information obtained during searching^49^. It is possible that pilot whales conduct similar assessment of prey layers and use prior information to inform their foraging decisions, or they could be more reliant on conspecifics, by eavesdropping or intentional information sharing on prey.

In conclusion, our analysis shows that diving behaviour in this species is more complex than a simple dichotomy of deep and shallow diving states. The variation we observed in diving behaviour may be driven by a number of factors including foraging success, prey availability and selection, bathymetry, physiological constraints and socially mediated behaviour. Individual short-finned pilot whales appear able to adapt their diving strategy on a dive by dive basis, switch effectively between different diving states, whilst maintaining foraging efficiency and social cohesion.

## Methods

### Data Collection

We equipped 20 short-finned pilot whales off Cape Hatteras, North Carolina, USA, with non-invasive, Version 2 DTAGs^3^ between 2008 and 2014. Tagging was performed from a variety of small rigid-hull inflatable vessels (all less than 10 m) in variable sea states (Beaufort 0–4), using a carbon-fibre pole to attach the tag to the dorsal surface or fin of the whale. The DTAG is a multi-sensor tag, attached via suction cups that records: audio with 16-bit resolution at a sampling rate of 96–192 kHz; depth at 50 Hz; and orientation of the whale from tri-axial accelerometers and magnetometers at 50 Hz^3^. The tags were programmed to release after a predetermined period, if they had not already detached from the animal, and were located (after floating to the surface) using a VHF radio transmitter embedded in the tag. In general this predetermined period was between 4 to 6 hours, to maximise chances of tag retrieval. However, in five instances longer predetermined periods were specified (Table 1).

In general, we selected an animal with distinctive natural marks in its dorsal fin, in a discrete group as the animal for tagging. Prior to tagging, photographs of the dorsal fins of all individuals within the group were taken for identification purposes. We avoided groups containing neonates as a condition of our permit. After tagging, we maintained non-systematic visual observations of the tagged animal and its group. These visual observations continued for the entire duration the tag was on the animal, unless periods of poor visibility (including during night time) rendered this impossible, or if animals were temporarily lost from view. For tag attachments that continued during night time, we tracked animals remotely from a large support vessel; the R/V Stellwagen (a 70 foot independent research vessel operated by Ocean Works Group, Inc.) using the VHF radio transmitter embedded in the tag. We obtained biopsy samples (see ref. 50 for methods) from eleven of the whales (typically immediately following release of the DTAG) and determined sex (see ref. 51 for methods) for nine of these individuals (Table 1). A quantitative analysis of the effects of tagging on our short-finned pilot whales showed no evidence of disruption of foraging behaviour and only low intensity responses to biopsy sampling^50^.

### Data Processing

Data were downloaded from the tags and pressure recordings were converted to depths using calibration information for each tag^3^. Calibration of the orientation offset from tag position was also performed and all movement data were down-sampled to 5 Hz. We followed Aguilar de Soto *et al*.^8^ and defined a *dive* as any submergence to a depth of 20 m or deeper. Any interval of data recorded at a depth of less than 20 m was considered time spent at the surface. Dive start and end times were determined by visual inspection of the dive profile. Each dive was considered as one sampling unit within a time series for each whale. We calculated three dive parameters for each dive: *Dive duration*, the time between the start of descent and the end of ascent (minutes); *Maximum depth*, the maximum depth reached during the dive (meters); *Number of buzzes*, the number of terminal echolocation click trains recorded during the dive. For pilot whales off Cape Hatteras, NC, USA, dive duration and maximum depth have been shown to be the two most important predictors of foraging dives with kinematic variables such as overall dynamic acceleration or average speed of movement showing no strong pattern with depth (M. Bowers 2016, Unpublished PhD Thesis, Duke University). Each parameter was calculated over the period of one dive (from time at surface when dive began to time when animal returned to the surface), using custom written code in Matlab (version 2014a). We removed incomplete data from any dive during which the tag detached from the whale. All acoustic audits of the DTAG sound files, to determine the start time and duration of buzzes, were completed by a single experienced analyst using custom written scripts for the DTAGs (available at http://soundtags.st-andrews.ac.uk) in Matlab version 2014a.

### Statistical Analysis

We used a multivariate hidden Markov model (HMM) as a framework for the analysis. The HMM allows unsupervised classification of dives into the most likely underlying, or ‘hidden’, state sequences that gave rise to our observations.The model involved an observed state-dependent process and an unobserved first-order N-state Markov chain that assumed the probability of being in the current state is determined only by the previous state^52^,^53^. The three dive parameters were specified as the observable series and were each assumed a distribution with state-dependent mean and variance parameters. The multi-state HMM considered the three observed dive variables as independent of each other, conditional on the 1^st^ order sequence of hidden states^23^,^54^. Dive duration and maximum depth were assumed Gamma distributions as they were continuous positive values, while the number of buzzes was assumed a Poisson distribution to allow these data to be treated as integer counts. Models were constructed based on two, three or four underlying non-observable behavioral states and that the observations were conditionally independent given the states, i.e., contemporaneous conditional independence was assumed^53^. We did not consider any higher state models. Increasing the number of states would result in a quadratic increase in the number of parameters and lead to a highly multimodal likelihood function, an unstable model and would render the model essentially uninterpretable biologically^24^,^25^,^53^.

Ten models were constructed. For three of the models we did not consider individual random effects, and assumed all whales shared common distribution parameters for all variables^23^. For the remaining seven models we incorporated discrete-valued random effects incorporated into the transition probability matrix^25^,^55^ to relax the assumption of homogeneity between whales (Supplementary Table S1). We assumed a transition matrix where all state transitions were possible and we included all dives from all individuals in the models. We initially also incorporated variation in heading and ascent and descent rate parameters into our models. None of these parameters had any effect on state allocations so were not considered further.

We fitted the models via numerical maximum likelihood estimation using the nlm optimizer in R^56^ (see ref. 53 for details of implementation). To improve confidence that the global maximum was found during the maximization process, we specified 100000 initial values and investigated the likelihood surface prior to maximization. This enabled only those values with the highest likelihoods to be passed to the nlm optimizer for maximization. One hundred runs of the models were completed to check for numerical stability in robustness against different initial values in the negative log likelihood. We applied the Viterbi algorithm^57^ to each individual animal and used it to find the most likely sequence of hidden states given the likelihood of the three observed variables under the estimated state-dependent distributions and the transition probabilities between states. We also calculated the frequency of appearance of each state and compared this to what would be expected if all states appeared evenly.

Finally, we conducted a linear regression, with tag duration as the response variable, to determine if there was a significant relationship between duration of the tag record and the proportion of time spent at the surface and a multiple regression between the duration of the tag record and the number of states, with one state as the reference level, in R software^56^. We computed the power of both regression models within the package pwr in R software^56^.

### Ethics Statement

All research activities were carried out under NOAA Permit 1421-03, issued to Peter Tyack; NOAA Permit 779-1633, issued to Keith Mullin; and NOAA General Authorization 16185, issued to Andrew Read, in accordance with the relevant guidelines and regulations on the ethical use of animals as experimental subjects. The research approach was approved by the Institutional Animal Use and Care Committee of Duke University.

## Additional Information

**How to cite this article**: Quick, N. J. *et al*. Hidden Markov models reveal complexity in the diving behaviour of short-finned pilot whales. *Sci. Rep.* **7**, 45765; doi: 10.1038/srep45765 (2017).

**Publisher's note:** Springer Nature remains neutral with regard to jurisdictional claims in published maps and institutional affiliations.

## Supplementary Material

Supplementary Information

## Figures and Tables

**Table 1:** Summary of tagging information, including the number of dives assigned to each state.

Date	Tag ID	Time on (local)	Time off (local)	Total Time (hh:mm)	Biopsy	Sex	Total dives	Number of dives per state			
								1	2	3	4
22-May-08	143a	14:28	17:41	03:13	N	—	4	2	2	0	0
22-May-08	143b	18:18	22:38	04:20	N	—	9	3	0	6	0
26-May-08	147a	15:02	17:20	02:18	N	—	7	7	0	0	0
30-May-08	151a	08:46	09:51	01:05	N	—	3	1	1	1	0
30-May-08	151b	13:14	05:56	16:42	N	—	17	9	1	7	0
04-Jul-10	185b	14:30	20:18	05:48	Y	F	5	1	3	1	0
05-Jul-10	186a	11:10	11:36	00:26	N	—	2	2	0	0	0
05-Jul-10	186b	14:32	16:28	01:56	Y	F	3	3	0	0	0
06-Jul-10	187b	12:53	06:07	17:14	Y	M	34	2	18	13	1
27-Jul-10	208a	14:50	23:30	08:40	Y	F	34	9	0	25	0
28-Jul-10	209a	08:54	10:09	01:09	Y	M	3	0	1	2	0
28-Jul-10	209c	13:19	19:45	06:26	Y	M	4	1	0	3	0
23-Sep-10	266a	18:35	12:49	18:14	N	—	32	3	11	4	14
24-Sep-10	267a	15:19	09:03	17:44	Y	M	64	7	1	43	13
29-May-11	149b	10:33	14:52	04:18	Y	M	7	1	6	0	0
30-May-11	150b	11:11	13:51	02:39	N	—	7	2	4	1	0
05-Jun-11	156a	12:11	15:07	02:55	N	—	3	0	0	3	0
14-Jun-11	165a	09:25	12:34	03:08	Y	M	9	0	7	2	0
07-Oct-14	279a	12:42	15:51	03:10	Y	U	10	5	2	0	3
08-Oct-14	280a	12:31	15:12	02:41	Y	U	2	1	0	1	0

Tag ID is based on the Julian day with the letter representing the sequential order in which the animal was tagged (a = first animal tagged that day, b = second and so forth). Biopsy, Y is a biopsy obtained, N is no biopsy obtained. Sex was obtained from the biopsy data, M = male, F = female, U = unknown (sample not processed), — = no biopsy taken. Total dives indicates the number of dives used per individual in the analysis. Number of dives per state shows the allocation of dives from the HMM.

**Table 2:** Transition probabilities for all states and number of dives within each state.

	State 1	State 2	State 3	State 4	Number of dives
State 1	0.526	0.168	0.275	0.030	59
State 2	0.040	0.714	0.134	0.112	57
State 3	0.142	0.087	0.730	0.041	112
State 4	0.069	0.148	0.140	0.643	31
