# Supplementary material for: Hidden Markov models reveal complexity in the diving behaviour of short-finned pilot whales
Source: Sci Rep. 2017 Mar 31;7:45765. doi: 10.1038/srep45765 (PMC5374633; doi:10.1038/srep45765)
Supplement: Supplementary Information [file srep45765-s1.doc]

Hidden Markov models reveal complexity in the diving behaviour of short-finned pilot whales.

Nicola J. Quick^1*^, Saana Isojunno^2^, Dina Sadykova^3^, Matthew Bowers^1^, Douglas P. Nowacek^1^ and Andrew J. Read^1^.

^1^ Division of Marine Science and Conservation, Nicholas School of the Environment, Duke University, Beaufort, North Carolina 28516, U.S.A.

^2^ School of Biology, University of St Andrews, Bute Building, St Andrews, Fife KY16 9TS United Kingdom

^3^ Zoology School of Biological Sciences, University of Aberdeen, Tillydrone Ave, Aberdeen, AB24 2TZ, United Kingdom

* Corresponding author: njq@duke.edu

**Supplementary Information**

**Supplementary Table S1**: Model selection criteria for all models. Mllk gives the negative log-likelihood score

| Model | AIC | BIC | Mllk |
| --- | --- | --- | --- |
| 2states_1cluster | 3077.375 | 3184.079 | 1508.687 |
| 2states_2clusters | 3083.78 | 3201.156 | 1508.89 |
| 2states_3clusters | 3059.614 | 3191.217 | 1492.807 |
| 3states_1cluster | 2548.965 | 2730.363 | 1223.483 |
| 3states_2clusters | 2581.345 | 2791.197 | 1231.672 |
| 3states_3clusters | 2585.809 | 2827.842 | 1224.905 |
| 3states_4clusters | 2576.812 | 2848.161 | 1211.406 |
| **4states_1cluster** | **2308.822** | **2579.141** | **1078.411** |
| 4states_2clusters | 2362.974 | 2692.799 | 1090.487 |
| 4states_3clusters | 2346.118 | 2733.054 | 1066.059 |
|  |  |  |  |

**Supplementary Table S2:** Model outputs from the multiple regression using 1 state as the reference level.

| **Variable** | **Estimate** | **Standard Error** | **p-value** |
| --- | --- | --- | --- |
| Intercept | 113.8 | 106.2 | 0.300 |
| 2states | 140.6 | 130.0 | 0.296 |
| 3states  4states | 239.0  950.2 | 142.4  162.2 | 0.113  <0.001 |


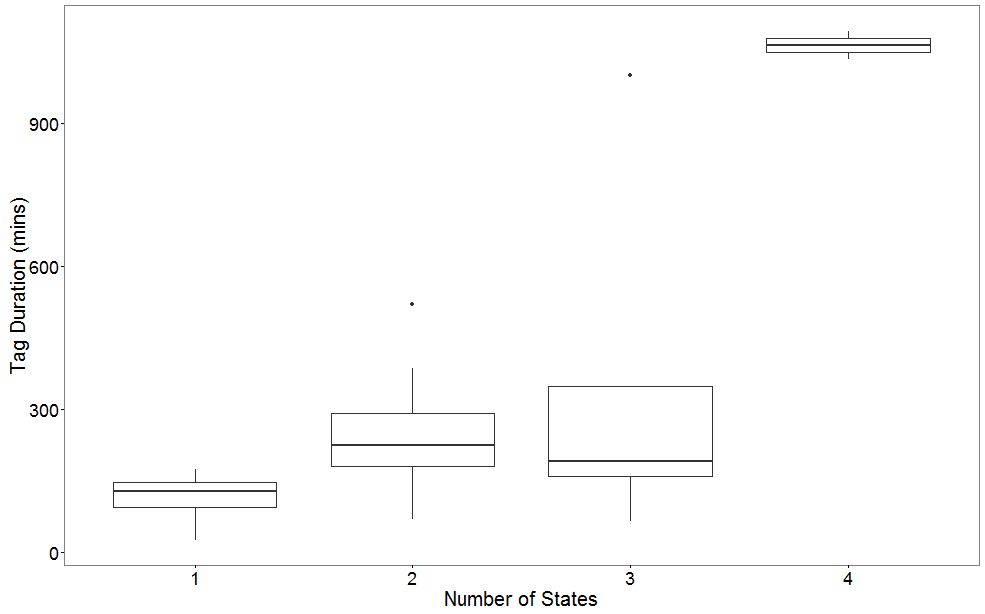


**Supplementary Figure S1**: Box plot of tag duration against number of states displayed.
